# Thermal Conductivity in Nanoporous Aerogels: A Critical Review of Gas and Solid Conduction Models and Structure-Property Relations

**DOI:** 10.3390/gels12040334

**Published:** 2026-04-17

**Authors:** Rajesh Ramesh, Murat Barisik

**Affiliations:** 1 Department of Mechanical Engineering, University of Tennessee at Chattanooga, Chattanooga, TN 37403, USA; rajesh-ramesh@utc.edu; 2 UTC Nanoengineering Center, University of Tennessee at Chattanooga, Chattanooga, TN 37403, USA

**Keywords:** aerogels, nanoporous materials, thermal conductivity, gas-solid coupling, Knudsen effect, structure-property relationship, thermal insulation, effective thermal conductivity

## Abstract

Sol–gel processing provides an unusually controllable route to nanoporous solids, making silica aerogels the leading reference systems for extremely low thermal conductivity due to their high porosity, nanoscale pore sizes, and tunable solid frameworks. Under near-ambient conditions, thermal transport is multi-scale and multiphase, arising primarily from coupled solid conduction through the skeletal network and gas conduction within the pore space. Accordingly, aerogel design has emphasized suppressing solid-phase transport by reducing network connectivity, increasing tortuosity, and enhancing boundary scattering, while also limiting gaseous conduction through the control of pore size and gas pressure. This critical review provides an integrated overview of these mechanisms and the theory-to-experiment toolbox used to quantify the separate and combined contributions of the solid and gas phases to the effective thermal conductivity. We link key structural and environmental parameters (porosity, pore size distribution, density, backbone morphology, and pressure) to dominant transport regimes and the assumptions embedded in common models. Classical approaches, including effective-medium and percolation-based models, are assessed alongside phonon-scaling descriptions that incorporate characteristic length scales. Particular attention is given to the Knudsen effect and pressure-sensitive gas-conduction models, which are central to interpreting performance at atmospheric conditions and under vacuum or low-pressure operation. This review highlights inconsistencies across datasets and modeling practices, identifies persistent knowledge gaps, and outlines practical directions toward processable structure–property guidelines for manufacturing aerogels with targeted thermal performance, with regard to conduction-dominated heat transport mechanisms.

## 1. Introduction

Thermal insulation is crucial for saving energy worldwide, since a lot of the energy we use is lost as unwanted heat from buildings, factories, and cold storage systems. Out of all the new insulation materials, nanoporous aerogels stand out for their ability to block heat flow better than almost anything else. Silica aerogels are more than 90% empty space, and their pores (just 10 to 100 nanometers across) and their fragile, web-like solid backbone act together to impede heat flow through virtually every available pathway. This unusual combination allows aerogels to suppress conduction through both the solid and the gas phase, as well as radiative transfer. Yet, despite decades of research, there has not been a coherent predictive framework. Some models overestimate solid-phase conduction, while others mischaracterize gas conduction, especially when confined in tight spaces, and radiative transport is treated inconsistently across the literature. Recent studies have explored data-driven and hybrid physics-informed approaches to predict thermal transport [1] and conductivity in aerogels [2,3,4], although their physical interpretability remains limited. Combining all these requirements into a single, reliable framework is still an open problem.

Nanoporous aerogels [5,6,7,8,9,10] are some of the best insulator materials available today. Recent advances have further expanded aerogel research into hierarchical [11], fibrous [12], and hybrid [13] architectures with enhanced thermal insulation performance, and wettability that depends on the volume of water droplets in contact with their surfaces [14]. They are exceptionally good at stopping heat from propagating, thanks to their super-high porosity, tiny pores on the nanometer scale, and a solid structure that is a tortuous, interconnected network. All these features work together to block heat, whether conducted through the solid phase, gas phase, or radiative transfer. Because we always need better ways to save energy, researchers are actively investigating exactly how these unusual materials block heat and how we can make them even better.

The thermal conductivity of porous materials can generally be expressed in the following manner:

(1) $$\kappa_{eff}=\kappa_{s}+\kappa_{g}+\kappa_{r}+\kappa_{c}$$

where the solid conduction through the aerogel backbone is represented by $\kappa_{s}$; $\kappa_{g}$ is the gaseous conduction through the pore space, $\kappa_{r}$ represents radiative heat transfer, and $\kappa_{c}$ stands for the gas–solid coupling effects arising from interactions between gas molecules and the solid framework. At near-ambient conditions, heat transport is often dominated by either the solid matrix or the gas phase. However, in nanoporous aerogels, the situation is more complex as it involves the complexity of the characteristic pore dimensions (typically 10–100 nm), which approach the mean free path of gas molecules (~60–70 nm for air at room temperature) [15,16,17]. Gas permeability is a factor in thermal transport through nanoporous systems and can be increased through rarefaction [18,19,20]. Under these conditions, collisions of gas molecules with the pore walls are more frequent than with one another, thereby reducing the gaseous thermal conductivity through the well-known Knudsen effect. This phenomenon is one of the main reasons as to why aerogels exhibit thermal conductivity values that are considerably lower than that of still air. A comprehensive investigation of gaseous conduction in nanoporous material systems at the molecular level has been done by Ghasemi and Barisik [21]. While the decomposition of the effective thermal conductivity in Equation (1) is widely adopted, it is rather a literature convention and not a physically independent decomposition. The individual terms are not uniquely measurable and lack strict independence in nanoporous aerogels, especially under atmospheric conditions, where confinement and gas–solid interactions occur together rather than in isolation.

**Figure 1:**
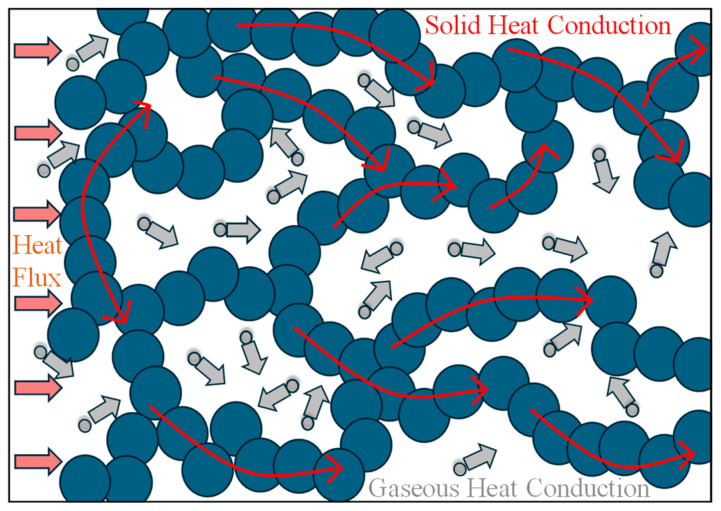
A pictorial representation of a silica aerogel structure showing the different mechanisms of heat conduction in it. The red arrows represent heat conduction through the solid part of the aerogel, and the gray arrows represent the gaseous heat conduction. The off-red arrows represent the heat source direction.

In the early experimental studies, silica aerogels demonstrated the ability to achieve room-temperature thermal conductivities as low as 0.01–0.02 Wm^−1^ K^−1^, far below that of most conventional insulation materials [22,23]. Such results sparked extensive research into the mechanisms governing heat transport in highly porous nanostructures. The experimental work that followed revealed that the overall thermal conductivity of aerogels is controlled by a delicate balance between multiple interacting transport processes. Solid conduction occurs through the tenuous network of interconnected nanoparticles that form the aerogel skeleton, while gas conduction depends on the type of gas, pore size, and gas pressure. Radiative heat transfer becomes increasingly important at elevated temperatures or when the solid density is extremely low [24,25,26]. These mechanisms interact with one another to create a complicated thermal transport landscape, as illustrated in Figure 1, that is strongly dependent on the aerogel’s microstructure.

Over the past several decades, numerous theoretical and experimental models have been developed to describe heat transfer in nanoporous aerogels. Classical approaches typically treated the total conductivity as a simple superposition of independent solid and gas contributions. However, this assumption often proved insufficient when it came to highly porous materials, as demonstrated by later studies. Solid–gas coupling mechanisms, in particular, where heat transfer occurs through interactions between gas molecules and the solid skeleton, were observed to significantly affect the effective thermal conductivity [27,28,29]. These coupling effects become especially important when pore sizes approach the molecular mean free path, leading to deviations from classical continuum heat-transfer models. As a result, several theoretical frameworks, such as Knudsen-based gas conduction models, effective medium theories, fractal and percolation models, and pore-network simulations, have been proposed to describe thermal transport in aerogels [16,30,31,32]. In spite of the progress so far, the accurate prediction and control of thermal conductivity in nanoporous aerogels continue to pose challenges. The complex hierarchical structure of these materials is a major contributor in this regard. The properties of aerogels include broad pore size distributions, fractal-like solid networks, and heterogeneous connectivity, making it difficult to characterize them using a single characteristic length scale that governs heat transfer [33,34,35,36]. In addition, experimental measurements of thermal conductivity considerably depend on the measurement technique, sample preparation, and environmental conditions. These uncertainties complicate the validation process of theoretical models and the establishment of viable structure–property relationships. Another key challenge lies in identifying the most effective strategies for engineering aerogels with even lower thermal conductivity. While early research primarily focused on reducing density and increasing porosity, it has become increasingly clear that microstructural design parameters such as pore size, particle connectivity, skeletal thickness, and hierarchical architecture play equally important roles in determining thermal transport behavior. Advances in synthesis techniques, such as sol–gel processing [37], freeze casting [38,39], templating approaches [40,41], and additive manufacturing methods [42,43], have enabled more precise control over aerogel microstructures and over aerogel thermal transport through deliberate design.

In recent years, different aerogel systems, such as hybrid and composite systems incorporating fibers, opacifiers, polymers, and nanostructured additives, have been investigated, with additives to further suppress heat conduction. Recent advances have also extended aerogel design beyond nanoparticle-based networks to include fibrous, nanosheet-based, and hierarchical architectures. The aim of these modifications is to decrease radiative heat transfer, disrupt solid conduction pathways, or modify gas transport within the pores. In parallel, emerging computational methods, including molecular dynamics simulations [44,45,46], lattice Boltzmann modeling [47], and pore-network analysis [48], have facilitated the understanding of nanoscale heat-transfer mechanisms, which are often inaccessible through experimental methods alone. Based on the literature so far, there is a clear need for a critical synthesis that integrates the diverse experimental observations, theoretical models, and structural design principles that govern thermal transport in nanoporous aerogels. The lack of understanding of the interaction of different mechanisms across multiple length scales, from molecular gas–solid interactions to macroscopic heat conduction, remains to be resolved in order to guide the development of next-generation ultra-low-conductivity materials. The discrepancy between the theoretical predictions and the experimental results is still prominent despite decades of research. These gaps are not just a reflection of the limitations of the existing models, since a significant part of the problem stems from how experiments are conducted and reported in the first place. Across the aerogel literature, there are inconsistent methodologies that create significant challenges in enabling meaningful comparisons of thermal conductivity data [49].

This review, therefore, provides a comprehensive and critical examination of the mechanisms, models, and design strategies underlying ultra-low thermal conductivity in nanoporous aerogels. The discussion begins with the fundamental heat-transfer mechanisms in aerogels, including solid conduction, gas conduction, and gas–solid coupling. This is followed by a review and critical evaluation of the major theoretical and computational models that describe these processes. Finally, key structure–property design parameters, such as pore-size engineering, hierarchical architecture, and compositional modification, are evaluated and scrutinized in the context of emerging approaches to design engineering aerogels. Through this integrated perspective, this review aims to clarify the current understanding of thermal transport in nanoporous aerogels and identify viable pathways for designing the next generation of superinsulating materials.

While existing reviews [50,51,52] have made important contributions to understanding thermal conductivity in aerogels, including modeling approaches for individual transport mechanisms and systematic characterizations of silica-based systems, a unified treatment that addresses gas–solid coupling as a primary topic in its own right remains limited. Most prior works discuss coupling effects as a secondary consideration within broader frameworks, without dedicated experimental and theoretical analysis. The present review fills this gap by treating solid, gaseous, and gas–solid coupling conductivity as distinct, equally important contributions, with each examined through both its theoretical basis and the experimental approaches used to probe it. It also points out the discrepancies associated with characterizing thermal conductivity using the density of aerogels. Furthermore, by extending coverage beyond silica aerogels to encompass a wider class of aerogel materials, this review enables cross-material comparisons that single-material-focused reviews are not positioned to offer. Together, these features define the distinct scope and contribution of the present work relative to the existing literature. Our review focuses mainly on the solid, gas, and gas–solid coupling conduction mechanisms in aerogels, especially under ambient conditions where the radiative thermal conduction is minimal.

## 2. Structural Characteristics of Nanoporous Aerogels

### 2.1. Three-Dimensional Nanoparticle Network Architecture

Aerogels derived from sol–gel processing exhibit hierarchical morphology. Primary silica particles, typically of 2–5 nm sizes, aggregate into chain-like structures that form a three-dimensional fractal backbone. Neck regions between particles form constrictions that act as thermal bottlenecks. The resulting network encloses a network of mesopores with diameters ranging from approximately 10 to 100 nm. The fractal dimension of silica aerogels commonly lies between 1.7 and 2.3, depending on synthesis and aging conditions. Hence, aerogels constitute a distinctive class of solid materials characterized by a highly interconnected three-dimensional (3D) solid framework surrounded by extensive air-filled pores. This structure spans from nanoscale constituents to macroscopic dimensions [53,54]. The nanoparticles of these materials randomly aggregate to form chain skeletons, which interconnect to form a complex three-dimensional network structure [51]. This network geometry is not merely structural in nature, but it is the structural origin of virtually every functional property the aerogel possesses, including its extraordinarily low thermal conductivity.

Based on their morphology, aerogels can be classified as particle-aggregated and fibrillar. Inorganic aerogels and some organic aerogels, such as resorcinol-formaldehyde (RF), melamine-formaldehyde (MF), and carbon aerogels, exhibit particle-aggregated morphology, while biopolymer aerogels, in particular, polysaccharides, show fibrillar morphology. Although particle-aggregated aerogels have similar network structures, they exhibit different characteristics; for instance, silica aerogels have fractal morphology due to the formation of bonds from nucleation and growth [55]. This fractal character has been confirmed by small-angle scattering studies, where small-angle neutron scattering (SANS) has been employed to characterize aerogel structures, examine fractal behavior at specific length scales, and study changes in structure with respect to processing variables [56].

### 2.2. Porosity, Pore Size Distribution, and Specific Area

**Figure 2:**
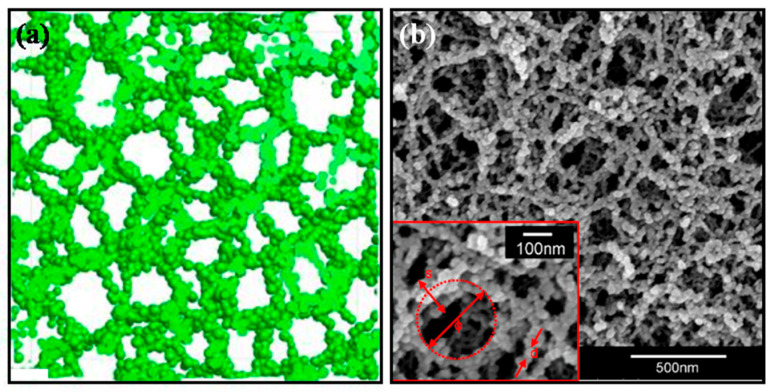
(**a**) Simulated pore network structure and (**b**) SEM images of silica aerogel. Adapted from open-access source [62].

In aerogels, heat conduction is governed by key structural parameters such as porosity, density, ligament diameter, pore size distribution, surface chemistry, and connectivity. Isolating their individual effects on thermal conductivity requires careful experimental and modeling approaches since these parameters are interdependent. This interplay between structural geometry and phonon physics is a key underlying factor as to why aerogels achieve thermal conductivities well below that of still air. Silica aerogels are defined by a set of distinct properties, including low density (0.003–0.5 g/cm^3^), large porosity (80–99.8%), low thermal conductivity (0.005–0.1 W/mK), an ultra-low dielectric constant (κ = 1.0–2.0), and a low refractive index (1.05) [57], all derived from their unique nanostructure consisting of a three-dimensional network of nanoparticles and submicron pores [58]. The pore network framework of silica aerogels has been illustrated in Figure 2. Most aerogel pores fall within the mesopore range, between 2 and 50 nm, though they often contain smaller micropores and larger macropores also. This complexity poses a significant challenge in determining the exact mean pore size and pore size distribution [56]. Accurate characterization of this distribution remains challenging. The applications of aerogels depend on their pore structure, but characterization of porosity is complicated by the compliance of the network. Standard methods such as mercury intrusion porosimetry (MIP), thermoporometry (TPM), and even nitrogen adsorption/desorption (NAD) can cause significant compression of the aerogel, leading to underestimation of pore volume and pore size [59]. The BET (Brunauer–Emmett–Teller) [60,61] method remains the standard for specific surface area measurement, while the Barrett–Joyner–Halenda (BJH) [61] method is widely used for mesopore size distribution, though BJH-derived pore sizes of 10–16 nm between samples are often smaller than calculated mean pore dimensions of 25–64 nm from geometric models, since BET analysis primarily reflects surface area contributions from mesopores and smaller macropores [56].

### 2.3. Density, Skeletal Connectivity, and Tortuosity

Two key parameters are commonly used to characterize silica aerogels: bulk density and skeletal density. Bulk density is the mass-to-volume ratio of the aerogel, ranging from 3 to 300 kg/m^3^, while the latter, which is the density of the solid framework itself, is approximately 2200 kg/m^3^, compared with the air density of approximately 1.2 kg/m^3^ [63]. The large disparity between these two values reflects the fact that the solid phase constitutes only a small fraction of the total volume. Accordingly, porosity is defined as

(2) $$\phi =1-\frac{\rho_{b}}{\rho_{s}}$$

where $\rho_{b}$ is the bulk aerogel density and $\rho_{s}$ is the skeletal density. Beyond porosity, the spatial connectivity and tortuosity of the solid backbone critically govern thermal transport. Models for solid conduction through aerogel skeletons must account for the tortuosity of the skeleton as well as clusters that are not connected to the solid network. These clusters do not contribute to the heat transfer. The contact area between secondary nanoparticles and the overall porosity of the secondary aerogel nanoparticles significantly influence the aerogel microstructure for a given density and, thereby, impact the total thermal conductivity of silica aerogels [64]. These structural descriptors, such as the nanoparticle size, neck contact area between adjacent particles, and network tortuosity, therefore, serve as key design levers. As the solid framework decreases in size to the nanoscale, phonon scattering increases, leading to a substantial reduction in solid-state thermal conduction, a phenomenon known as the size effect in the solid structure of aerogels [65].

### 2.4. Beyond Nanoparticle-Based Aerogels: Emerging Architectures

While classical aerogels are typically formed from aggregated nanoparticles (0D building units) arranged in necklace-like fractal networks, recent advances in synthesis have enabled a broader range of aerogel architecture based on different dimensional building blocks. These include one-dimensional (1D) nanofiber-based aerogels, such as cellulose [66], polymer [67,68], and ceramic [69] nanofiber networks, two-dimensional (2D) nanosheet-based aerogels, including graphene [70] and MXene [71] assemblies, and hierarchical composite structures [72] that combine multiple length scales and building-unit types. These emerging architectures exhibit distinct structural characteristics compared to traditional particle-based aerogels. Fibrous aerogels typically display higher connectivity and anisotropic networks with reduced thermal constriction resistance along fiber pathways, while nanosheet-based aerogels introduce layered structures with enhanced scattering and interfacial resistance. Hierarchical and hybrid systems further enable independent tuning of pore size distribution, connectivity, and mechanical properties across multiple length scales. From a thermal transport perspective, these structural differences significantly influence heat conduction mechanisms. One-dimensional networks can reduce phonon scattering along continuous pathways while increasing tortuosity at larger scales, whereas two-dimensional assemblies enhance interfacial scattering and can modify radiative transport through increased absorption and scattering of infrared radiation. These effects highlight that thermal conductivity in aerogels cannot be understood within a single structural paradigm, but must instead be interpreted within a broader, structure-dependent framework that encompasses multiple architectural classes.

### 2.5. Anisotropic Aerogels and Directional Thermal Transport

In addition to isotropic nanoparticle-based aerogels, recent developments have enabled the fabrication of anisotropic aerogels with directionally aligned pore structures, often inspired by natural materials such as wood [73,74,75]. These structures are commonly produced using techniques such as freeze casting [39,76], directional solidification [77], or templating [78,79], resulting in lamellar or channel-like architectures with distinct axial and transverse directions. Such anisotropic architectures give rise to strongly direction-dependent thermal conductivity. In the direction parallel to aligned channels or fibers, heat can propagate more efficiently due to enhanced connectivity, whereas in the perpendicular direction, increased tortuosity and scattering lead to significantly reduced thermal conductivity. As a result, anisotropic aerogels can exhibit exceptionally low thermal conductivity in one direction while maintaining structural integrity in another.

## 3. Heat Transfer Behavior and Related Mechanisms in Aerogels

Understanding how heat moves through a nanoporous aerogel requires disentangling several co-existing mechanisms, with each operating at different length scales and responding differently to structural and environmental variables. In most cases, heat transfer within non-evacuated aerogels is based on three mechanisms: heat conduction via the solid backbone, heat transfer within the gaseous phase present in the open-porous aerogel structure, and radiative heat transfer [51]. Heat convection in pores can be neglected when the pore size is smaller than 1 mm, and since aerogel pore sizes are mainly in the order of nanometers to tens of nanometers, convective contributions are effectively absent [80]. As mentioned in Equation (1), the effective total thermal conductivity is a combination of solid, gaseous, solid–gas coupling, and radiative contributions to the thermal conductivity. Radiative contribution to thermal conductivity, which becomes especially significant at high temperatures (>1000 °C), can be eliminated or minimized by making the material opaque to infrared radiation, incorporating reflective barriers, or introducing scattering centers. Effective methods include adding infrared blockers (such as graphene nanoplatelets or metal oxides), applying reflective low-emissivity coatings, or using vacuum layers to suppress radiative heat transfer [81,82]. In the simplest case, all heat transfer mechanisms are treated as independent of each other and described using a diffusion model, which defines specific thermal conductivities for each mechanism according to Fourier’s law [83].

Determining the individual contributions of each mode is a long-standing challenge, since they cannot be cleanly separated in a single measurement. The community has converged on four broad methodological pillars for this purpose. The first is experimental measurement, which provides direct, macroscopic thermal conductivity values. There are two main groups of experimental methods: steady-state and transient methods. Stationary methods, such as the guarded-hot-plate and heat-flow-meter methods, subject a specimen to a temperature gradient and measure temperatures at the external boundaries, the specimen thickness, and the heat flow to determine conductivity [57]. Transient methods, including the hot-strip method, transient hot wire technique, transient plane source (TPS) method, and laser-flash method, have also been employed to investigate effective thermal conductivity, particularly at temperatures below 1000 °C [84]. Each mode’s contribution can be isolated experimentally by varying pressure (to suppress gaseous conduction), temperature (to amplify radiation), or composition (to alter solid-phase geometry), and comparing outcomes against a baseline. The second pillar is theoretical modeling, which derives closed-form or semi-analytical expressions for each conductivity component from first principles, kinetic gas theory, phonon transport theory, and the radiative transfer equation. For gaseous conduction, three broad theoretical strategies have been identified: empirical correlation of measured gas conductivity against material density; numerical simulation via the Lattice Boltzmann Method (LBM) [85,86], Direct Simulation Monte Carlo (DSMC) [87], or Molecular Dynamics (MD); and derivation from molecular motion theory to obtain gas thermal conductivity in nanoscale pores [88]. The third pillar is empirical/continuum modeling, which calibrates semi-empirical expressions for each contribution against experimental datasets and then embeds them within effective medium or unit-cell frameworks to predict bulk conductivity. Finite element methods using continuum modeling can predict the characteristics and functional behavior of an aerogel based on its aerogel-forming structure, and simulation can also be used to design the pore structure of aerogels and evaluate characteristics under various environmental conditions [56]. The fourth and increasingly prominent pillar is atomistic simulation, chiefly classical molecular dynamics (MD), which directly resolves heat transport at the nanoscale without relying on bulk material properties. MD simulation, based on classical mechanics with limited assumptions, has been used to reveal microscopic aspects of solid–gas coupling heat transfer in aerogels, and plays an important role in improving the prediction of thermal conductivity; it can be applied alone or as part of multi-scale and integrated simulations [89]. Radiative heat transfer increases progressively with temperature and may contribute appreciably even at moderate temperatures in optically semi-transparent aerogels [25], with its relative importance governed by spectral transparency, pore structure, and the presence of opacifying additives. Radiative heat transfer [90,91,92], while important particularly at elevated temperatures, very low densities (high porosity), and in materials with high infrared transparency, is not treated in detail in this review, as the focus is placed on conduction-dominated mechanisms and gas–solid coupling effects under near-ambient conditions.

### 3.1. Solid Thermal Conductivity in Aerogels

Solid thermal transport in aerogels occurs through a highly tortuous, low-density silica backbone. Classical effective medium theories treat the material as a two-phase composite of solid and gas. However, at porosities exceeding 90%, simple mixing rules fail to accurately capture the drastic suppression of conductivity. Heat conducted through the solid backbone travels along tortuous nanoparticle chains and is scattered at every neck contact and surface. When heat is transferred through the solid skeleton, the complex skeleton structure of nanoporous insulation materials increases the heat transfer path, creating large thermal resistance, and thus the solid thermal conductivity is lower than that of bulk silica. This suppression arises from two compounding mechanisms. First, the geometrical tortuosity and slender contact necks between primary particles create a constricted, high-resistance pathway. Second, at the nanoscale, phonon mean free paths become comparable to or larger than particle and neck dimensions, causing boundary and interface scattering to dominate over bulk scattering. The nanoporous structure of aerogels impedes the movement of gas molecules, and the nano-skeleton system restricts solid heat transfer because of the size effect [93]. Percolation-based models [94] recognize that thermal transport depends strongly on connectivity. As porosity increases, the backbone approaches a percolation threshold, and conductivity scales according to power–law relationships. Fractal models [34,35,36] further refine this description by incorporating structural self-similarity, linking apparent conductivity to fractal dimension parameters measured via scattering techniques. At the nanoscale, phonon-boundary scattering becomes dominant. When ligament diameters approach or fall below phonon mean free paths, classical Fourier conduction assumptions break down. The effective phonon mean free path is reduced due to boundary scattering at neck regions, leading to conductivity values significantly lower than density-scaled bulk silica predictions.

A variety of analytical and numerical models have been developed to predict solid-phase conductivity in aerogels, differing principally in their treatment of pore geometry, phonon scattering mechanisms, and the level of structural idealization. Table 1 gives an overview of the theoretical models involved in predicting the solid thermal conductivity in aerogels. Bauer [31] developed a closed-form effective-medium expression derived from field-perturbation solutions of Laplace’s heat conduction equation, using a pore-shape factor to accommodate spherical, fibrous, and plate-like pore geometries across a wide porosity range. Bi and Tang [95] came up with a model that takes a more physically explicit approach, representing the skeleton as a network of interconnected nanoparticles and computing heat flow through the thermal constriction resistance at particle–particle neck contacts, combined with phonon kinetic theory for the intrinsic backbone conductivity. Sumirat et al. [96] generated a purely analytical phonon-scattering model that modifies the classical kinetic theory expression $\kappa =\frac{1}{3}Cvl$ by applying Matthiessen’s rule [97] to account for additional pore-boundary scattering, introducing the dimensionless ratio $l_{0}/R_{p}$ as the governing parameter that controls the transition between volumetric dilution and strong size-effect regimes. The Fang and Pilon [98] model is anchored in non-equilibrium molecular dynamics (NEMD) simulations of porous silicon, from which a physics-based analytical scaling law is extracted that captures both pore-surface scattering, through the interfacial area concentration $A_{i}$, and boundary scattering through the system length $L_{z}$. Another theoretical model is the percolation model [94], which combines the Looyenga effective-medium framework [99] with phonon diffusion theory, replacing the phonon mean free path with the mean crystallite size $d_{k}$ and introducing a percolation connectivity factor that causes the effective conductivity to scale as the cube of the solid volume fraction. In contrast, the Glicksman model [100] is a continuum foam-geometry model that decomposes solid conduction into separate contributions from cell walls and struts, yielding a closed-form expression that depends on porosity, the material conductivity $k_{p}$, and the fraction of solid residing in struts versus walls.

**Figure 3:**
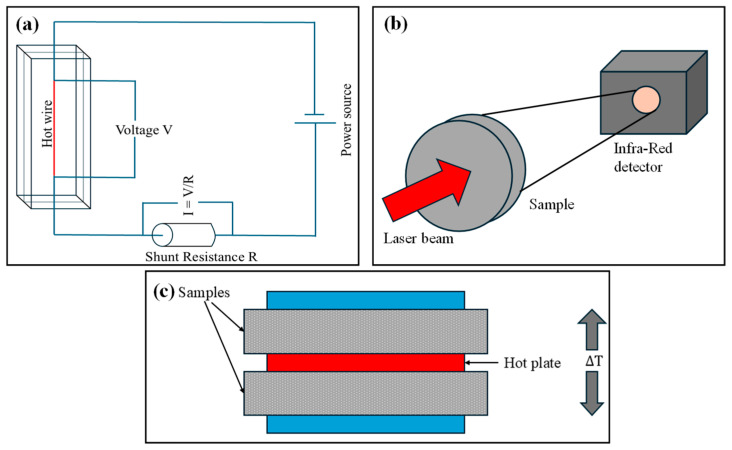
Experimental techniques used to determine the thermal conductivity of aerogels. (**a**) Hot-wire method, (**b**) laser-flash method, and (**c**) guarded hot-plate method. The blue plates represent the cold surface assembly on either side of the samples sandwiching the hot plate.

A principal challenge in characterizing the solid contribution to aerogel thermal conductivity is that it cannot be measured in isolation, and any real measurement captures an effective value that conflates solid conduction, gaseous conduction, and radiation simultaneously. Figure 3 shows a few of the experimental methods employed to determine the thermal conductivity of aerogels. Table 2 provides a rundown of the experimental techniques used to calculate the solid thermal conductivity in aerogels. The guarded hot plate method applied to silica aerogels [101] achieves this by operating under vacuum to eliminate gas conduction and incorporating opacifiers to minimize radiative transport, yielding effective conductivities of 0.003–0.025 W/mK across porosities of 89–96%, values that closely approximate $\kappa_{solid}$ alone, given the Knudsen suppression regime. An analogous guarded hot plate study on carbon aerogels [102] reports a broader effective conductivity range of 0.005–0.25 W/mK for porosities of 75–98%, exploiting the negligible radiative contribution of carbon at ambient temperatures to similarly isolate the solid term. For thin-film aerogel systems, where bulk measurement techniques are inapplicable, the 3ω method has been widely adopted. Applied to silica xerogels [103], it confirms the absence of pressure dependence in nanoscale pores, which is a hallmark of the Knudsen regime, giving solid conductivities across a porosity range of 25–80%. The same technique applied to MSSQ organosilicate porous films [104] subtracts substrate and oxide cap contributions to isolate the cross-plane solid conductivity, reporting values of 0.1–0.35 W/mK for porosities up to 50%. Time-Domain Thermoreflectance (TDTR) applied to mesoporous silica films [105] takes a complementary approach, intentionally removing the gas phase so that the TDTR signal directly represents the solid skeleton contribution, spanning 0.07–0.66 W/mK for porosities of 9–69%. Finally, a custom transient experiment on SiC-doped silica aerogels [106] separates the gas term explicitly using a phonon size-limited model, reporting effective solid conductivities of 0.05–0.09 W/mK for porosities of 70–90%. These effects may be further modified in emerging aerogel architectures based on fibrous or nanosheet building units, where connectivity and interfacial density differ significantly from classical nanoparticle networks.

The models discussed in this paper were developed primarily for silica aerogels, and their assumptions, fractal backbone geometry, amorphous neck constrictions, and phonon scattering dominated by inter-particle contacts reflect silica’s specific sol–gel derived microstructure. For carbon aerogels, the solid-phase conductivity is substantially higher due to the graphitic character of the backbone, and phonon transport is less constriction-limited than in silica. Fibrillar aerogels such as cellulose and polysaccharide-based systems present an entirely different morphology where the neck-constriction picture does not apply, and conductivity is instead governed by fiber–fiber contact resistance and fiber aspect ratio. The fractal and percolation models reviewed here, therefore, transfer only qualitatively to non-silica systems, and their quantitative use outside silica aerogels requires re-parameterization against system-specific structural data.

The description of thermal transport in anisotropic aerogel systems requires moving beyond scalar conductivity to a tensorial representation, where thermal conductivity is expressed as a second-order tensor with distinct components along principal directions. Effective medium models and network-based approaches can be extended to account for directional connectivity and anisotropic pore morphology, although predictive capability remains limited due to the complexity of real structures. An analytical approach to characterize the solid thermal conductivity of a cellulose-based anisotropic aerogel based on the phonon transport theory derived the solid thermal conductivity from the phonon mean free path [107]. Experimental characterization of anisotropic thermal conductivity [39,108] also requires direction-sensitive measurement techniques. Steady-state methods such as the guarded hot plate can be adapted to probe specific orientations, while transient techniques such as the transient plane source (TPS) method can provide directional information when properly configured. However, accurate measurement remains challenging due to contact resistance, alignment precision, and the need to ensure well-defined heat flow along principal axes.

**Table 1:** Theoretical models for the calculation of solid thermal conductivity of aerogels.

Model	How It Was Developed	Physical Parameters	Validation
**Bauer model** [31] $\kappa_{s}=\kappa_{0}{\left(1-\phi \right)}^{\frac{3}{3\epsilon -2}}$ ϵ=1 (spherical pores), ϵ>1 (fibrous/elongated pores) ϵ<1 (flattened/plate-like pores)	Closed-form effective-medium model derived from microscopic field perturbation theory via solutions of Laplace’s equation for heat conduction in heterogeneous media.	Solid parameters: $\kappa_{0}$ (Matrix conductivity), $\kappa_{s}$ (Solid-phase thermal conductivity) Pore parameters: ϕ (Porosity), $\kappa_{p}$ (Pore-phase conductivity), ϵ (Pore shape factor)	Validated against multiple independent literature datasets (liquid foams, packed/debris beds, fibrous materials) across wide porosity (0.2–0.95) and conductivity-ratio ranges (up to 10^4^–10^5^), using fixed geometry-based shape factor with no refitting.
**Aerogel solid backbone heat transfer model** [95] $\kappa_{p-p}=\frac{2\sqrt{r^{2}-a^{2}}}{R_{t}\pi r^{2}}$ r: Radius of the nanosphere $R_{t}$: The overall thermal resistance consisting of the thermal resistance of two hemispheres and constriction a: Contact radius	Physics-based analytical model of backbone thermal conductivity, built on the kinetic theory of phonon conduction in a 3D network of interconnected spherical nanoparticles with defined particle and neck diameters.	Material parameters: $C_{V}$ (Specific heat), $v_{\mathrm{bulk}}$ (Sound velocity), $\kappa_{\mathrm{bulk}}$ (Bulk thermal conductivity), $\rho_{0}$ (Atomic density) Structural parameters: ρ (Aerogel density), $d_{p}$ (Particle diameter), a (Contact diameter)	Validated against silica aerogel [101] and carbon aerogel [102] experimental datasets with no fitting to conductivity values; good agreement for densities above 100 kg/m^3^, correct density trends, and major improvement over bulk conductivity.
**Sumirat model** [96] $\kappa_{s}=\frac{\kappa_{0}\left(1-\phi \right)}{1+\frac{l_{0}}{R_{p}}\phi^{\frac{1}{3}}}$ $\kappa_{0}$: Bulk thermal conductivity (when ϕ = 0) $\kappa_{0}=\frac{1}{3}C_{0}v_{0}l_{0}$ $l_{0}$: Bulk mean free path $R_{p}$: Pore size ϕ: Porosity	Purely analytical phonon kinetic theory model for nanoporous materials, incorporating porosity effects on heat capacity (scales with solid fraction), and phonon mean free path via Matthiessen’s rule [97], combining intrinsic and pore-scattering contributions, with pore size explicitly modeled assuming randomly distributed isolated pores.	Material parameters: $C_{0}$ (Heat capacity of solid), $v_{0}$ (Phonon velocity), $l_{0}$ (Bulk phonon mean free path), $\kappa_{0}$ (Bulk solid conductivity) Structural parameters: ϕ (Porosity), $R_{p}$ (Pore size)	Validated against known limiting cases, Maxwell and percolation models, and experimental data from nanoporous silica and MSSQ films (pore size ≈ phonon mean free path ≈ 3–5 nm), with good agreement and no fitting parameters.
**Fang and Pilon model** [98] $\kappa_{s}=\frac{\frac{1}{3}C_{v,m}v_{g,m}\left(1-1.5\phi \right)}{\frac{A_{i}}{4}+\frac{1}{L_{z}}}$ $C_{v,m}$: Specific heat $v_{g,m}$: Group velocity of the Si matrix ϕ: Porosity $A_{i}$: Pore interfacial area $L_{z}$: System length	NEMD simulation study (Stillinger-Weber potential) generating effective thermal conductivity data for crystalline silicon with spherical pores in a simple cubic arrangement, combined with a physics-based analytical model integrating phonon kinetic theory, Matthiessen’s rule (pore and boundary scattering), and effective medium theory.	Morphological parameters: ϕ (Porosity), $d_{p}$ (Pore diameter), $A_{i}$ (Interfacial area concentration), $L_{z}$ (System length/film thickness) Material/phonon parameters: $C_{v,m}$ (Heat capacity), $v_{g,m}$ (Phonon group velocity), Phonon mean free path (implicit)	MD method validated against literature, experimental bulk Si conductivity, and finite-size scaling; analytical model validated by collapse of all MD data (varying porosity, pore size, and length) onto a single scaling curve, and externally against independent MD studies of cylindrical pores and vacancy defects across different potentials, temperatures, and geometries.
**Percolation model** [94] $\kappa_{s}=\frac{1}{3}f^{3}\rho C_{v}vd_{k}$ f=1−ϕ: Solid volume fraction ρ: Density $C_{v}$: Mass-specific heat capacity v: Sound velocity $d_{k}$: Mean crystallite size.	Combines 3ω [109,110] experimental measurement with a phonon-diffusion analytical model incorporating Looyenga effective medium theory [99], percolation-based connectivity, and phonon mean free path limitation by crystallite size rather than intrinsic scattering.	Structural parameters: ϕ (Porosity), f=1−ϕ (Solid volume fraction), $d_{k}$ (Crystallite size) Material/phonon parameters: ρ (Density), $c_{v}(T)$ (Specific heat), v (Sound velocity), l (Phonon mean free path, limited by $d_{k}$)	Validated experimentally via 3ω measurements across 35–320 K, porosities 64–89%, two doping levels, and multiple layer thicknesses; excellent agreement with model predictions for porosity dependence, temperature dependence, and doping effects via crystallite size.
**Glicksman model** [100] $\kappa_{s}=\frac{\kappa_{p}}{3}\left(1-\phi \right)\left(2-f_{s}\right)$ $\kappa_{p}$: Material conductivity $f_{s}=1-\phi$: Solid volume fraction ϕ: Porosity	Closed-form analytical continuum model decomposing foam conductivity into solid, gas, and radiative contributions, with solid conduction modeled separately for cell walls and struts using idealized foam geometries (cubic cells, staggered cubes, randomly oriented walls), validated against multiple independent experimental datasets.	Structural parameters: ϕ (Porosity), Cell size, Cell orientation, $f_{s}$ (Strut fraction), Cell wall thickness, a/b (Anisotropy ratio), Strut length and orientation distribution, Cell wall surface area per unit volume Material parameters: $\kappa_{p}$ (Polymer conductivity), Density of solid polymer, Solid fraction in struts vs. walls	Validated against electrical conductivity in aqueous foams [111], vacuum thermal conductivity of open-cell foams [112], anisotropic foam experiments, and crushed-foam polymer measurements; open-cell foam in vacuum case shows agreement within 2% between measured and predicted solid conductivity.
**Analytical model for cellulose-based aerogels** [107] $\kappa_{s}\approx \kappa_{bulk}\left(\frac{l_{eff}}{l_{bulk}}\right)$ $l_{eff}$: Effective phonon mean free path	Developed using a cellular nanofoam representation of the aerogel structure, where the solid phase is modeled via mean free path-based phonon transport theory combined with a phonon tracking approach, accounting for nanoscale size effects in the cellulose skeleton.	Structural parameters: $d_{p}$ (Cell size,) ϕ (Porosity), Solid fraction	Validated experimentally through comparison of predicted effective thermal conductivity with measured values under varying temperature and pressure conditions.

**Table 2:** Experimental techniques to capture the solid thermal conductivity of aerogels.

Experiment	Solid Contribution	Bulk Thermal Conductivity	Reported Range
**Guarded hot plate for silica aerogels** [101]	Evacuated, opacified, Knudsen-regime conditions isolating solid conduction contribution; measured effective conductivity directly compared to predicted solid conductivity.	1.3–1.4 W/mK	0.003–0.025 W/mK for porosity 96–89%
**Guarded hot plate for carbon aerogels** [102]	Gas and radiation contributions both negligible; measured effective conductivity isolates solid conductivity directly.	5–6 W/mK	0.005–0.25 W/mK for porosity 98–75%
**Thin-film study using 3ω method for silica xerogels** [103]	Nanoscale pores; no pressure dependence measured, confirming gas conduction negligible.	1.35 W/mK	0.15–1.25 W/mK for porosity 80–25%
**3ω method for MSSQ organosilicate porous films** [104]	Cross-plane gas conduction negligible; Si substrate and oxide cap contributions subtracted from measurements.	0.35 W/mK	0.1–0.35 W/mK for porosity 50–0%
**Time-Domain Thermoreflectance (TDTR) on mesoporous silica films** [105]	Gas intentionally removed; TDTR signal represents solid skeleton conduction only.	1.3–1.4 W/mK	0.07–0.66 W/mK for porosity 69–9%
**Custom transient experiment on SiC-doped silica aerogels** [106]	Gas contribution separated; phonon size-limited model applied to solid skeleton.	1.3 W/mK	0.05–0.09 W/mK for porosity 70–90%
**Anisotropic freeze-cast cooling aerogels engineered with Boron Nitride Nanosheets (BNNS) in waterborne polyurethane (WPU)** [39]	Inferred indirectly by combining total thermal conductivity measurements with structural control (porosity, alignment) and by minimizing gas (small pore size) and radiative (at room temperature) contributions.	In-plane BN: 200–400 W/mK Out-of-plane BN: 2–30 W/mK	In-plane: 0.05–0.1 W/mK Out-of-plane: 0.016–0.03 W/mK
**Top-down approach-fabricated wood aerogel** [108]	Gas conduction and radiation suppressed; interpreted via directional (parallel vs. perpendicular) measurements	~0.10 W/mK (radial) ~0.15 W/mK (axial)	~0.028 W/mK (perpendicular to fiber alignment) ~0.12 W/mK (parallel)

The selection of experimental techniques for measuring thermal conductivity in aerogels depends on several practical factors, including sample size, thermal conductivity range, structural anisotropy, and environmental control. Steady-state methods such as the guarded hot plate provide high accuracy and are well-suited for bulk samples, but require relatively large specimen sizes and long equilibration times. In contrast, transient methods such as the transient plane source (TPS, or hot disk) technique are widely used for nanostructured aerogels due to their ability to accommodate small samples and provide rapid measurements. However, these methods may be more sensitive to contact resistance, sample heterogeneity, and boundary effects. Heat flow meter methods offer a compromise between simplicity and accuracy but are typically calibrated for higher-conductivity materials and may be less reliable in the ultra-low conductivity regime. Consequently, method selection involves balancing accuracy, sample constraints, and experimental feasibility, and results obtained from different techniques should be compared with caution. The advantages and limitations of the different analytical models and the experimental techniques have been mentioned in Table 3.

Across nearly all models, there exists a drawback, which is the assumption of a single, representative pore size and a homogeneous solid fraction. Real aerogels exhibit broad pore size distributions, spatially varying density, and fractal network topology, all inaccurately captured by the existing analytical models. This is a major challenge in modeling solid thermal conductivity. Multi-scale frameworks that couple atomistic phonon transport at the particle and neck scale with continuum network descriptions at the mesoscale would need to be incorporated into future modeling efforts. Integrating the models with high-resolution structural characterization data, namely data from small-angle X-ray scattering (SAXS) and tomographic imaging, would result in realistic geometric inputs rather than idealized ones. Despite the considerable ingenuity reflected in the aforementioned experimental strategies, several important limitations and inconsistencies deserve critical attention. The most fundamental issue is that no experiment truly measures solid-phase conductivity in isolation, since each study relies on assumptions about the magnitude of the suppressed or subtracted contributions, and errors in those assumptions propagate directly into the reported solid conductivity values. In guarded hot plate measurements, for instance, achieving sufficiently low pressures to fully enter the molecular flow regime is technically demanding, and any residual gas conduction will cause the solid contribution to be systematically overestimated. The 3ω method is sensitive to interfacial thermal resistance between the film and substrate, even though it is compatible with thin films, and the subtraction of substrate contributions introduces its own uncertainty, especially when film thicknesses are small relative to the thermal penetration depth. TDTR offers excellent spatial resolution and does not require physical contact. But its accuracy depends critically on the thermal model used to fit the measured signal, and there could be systematic errors when the values of heat capacity and density are assumed. The comparability of reported values across studies is also a matter of concern, even with bulk density, skeletal density, pore size distribution, and synthesis route varying considerably between studies. Moreover, a single effective conductivity is reported by most experiments at ambient temperature, and this provides no information about the dependence of the solid contribution on temperature. This is an important gap, given that phonon scattering mechanisms and mean free path are inherently temperature-dependent. Future experimental work would benefit from standardized reporting protocols that include full structural characterization alongside thermal measurements, as well as systematic pressure-dependent and temperature-dependent measurements that would allow the individual contributions to be decoupled with greater confidence.

**Figure 4:**
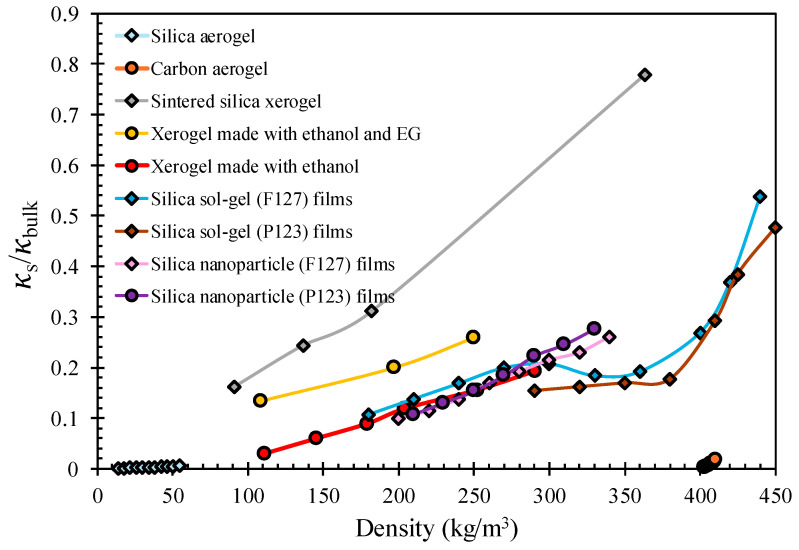
Experimentally measured and normalized thermal conductivity values plotted against the density of nanoporous and mesoporous materials. Data from [101,102,103,104,105,113]. Data compiled from multiple literature sources with differing measurement conditions; values are not strictly comparable and illustrate general trends.

As density increases, which corresponds to a reduction in porosity, the solid-phase conductivity approaches a larger fraction of the bulk value. In Figure 4, a few of the curves exhibit non-monotonic behavior that departs from this trend. For example, the sintered silica xerogel series shows an uncharacteristically steep rise in the normalized thermal conductivity value at relatively low densities. This is most probably a resultant effect of the sintering process, where solid connectivity is improved by the elimination of narrow inter-particle necks and the fusion of nanoparticles, at a higher temperature, leading to a dramatic reduction in the phonon constriction resistance. This illustrates a critical limitation of using density, and by extension porosity, as the sole structural descriptor. Two materials with identical densities can have vastly different conductivities depending on their neck geometry and thermal contact quality, a nuance that none of the purely density-based empirical models can capture. The decreasing or plateauing normalized thermal conductivity is interesting, since in dense, well-connected solids, conductivity is expected to increase monotonically with density. A local decrease instead suggests either a structural transition within the material, such as a change in pore morphology, network topology, or particle connectivity, or an experimental artifact such as increased interfacial resistance in thicker or denser films. Thus, there is a compelling case that density is a necessary, but insufficient, descriptor of solid thermal conductivity in nanoporous materials. Pore morphology, inter-particle neck geometry, material chemistry, and measurement methodology each exert independent and significant influences on thermal conductivity that are invisible to models that characterize only density. A truly predictive framework for solid-phase conductivity must therefore incorporate structural descriptors beyond density. What this entails, especially with regard to Figure 4, is that density and porosity are both necessary but not sufficient as entirely reliable parameters to describe solid thermal conductivity. The same density can yield vastly different conductivities depending on neck size, connectivity, tortuosity, pore-size distribution, and surface interaction parameters. A single-parameter description is insufficient, as evidenced by the non-monotonic U-shaped behavior in Figure 4. When different labs measure thermal conductivity on what should be essentially the same materials, the reported values can vary quite a bit. It is a pattern that keeps showing up in interlaboratory comparisons, and it speaks to a fairly fundamental issue, which is that there are still no widely standardized protocols for how these measurements should be done [49].

### 3.2. Gas Contribution to Thermal Conductivity in Aerogels

The gaseous thermal conductivity of aerogels is governed by the Knudsen effect, whereby the confinement of gas molecules within nanoscale pores suppresses molecular collisions and reduces the effective gas conductivity well below its free-gas value. A summary of the theoretical models involved in predicting the gaseous thermal conductivity in aerogels can be found in Table 4. The Kaganer model [30] is the foundational expression that takes the simple closed-form Knudsen correction:

(3) $$\kappa_{g}=\frac{\kappa_{g}^{0}}{1+2\beta Kn}$$

where $\kappa_{g}^{0}$ is the free-gas thermal conductivity, Kn is the Knudsen number defined as the ratio of the molecular mean free path to pore diameter, and β is a constant that encapsulates the thermal accommodation coefficient and the adiabatic coefficient of the gas. The expression in Equation (3) serves as the benchmark for all subsequent models. The mean free path is relevant in gas conduction since it is the result of gas–gas collisions and gas–wall collisions in nanoporous materials [114]. Zeng et al. [16,115] derived a gas kinetics model that takes a more detailed approach by deriving the gas conductivity analytically from molecular kinetic theory, incorporating the specific surface area S and aerogel density $\rho_{a}$ into the effective mean free path, thereby linking the suppression of gas conductivity directly to measurable structural properties of the aerogel rather than relying on an empirical β parameter. Reichenauer et al. [17] extended the Kaganer model by explicitly incorporating porosity ϕ as a prefactor and expressing the Knudsen number in terms of gas pressure relative to a reference pressure, which allows the model to be directly fitted to pressure-dependent conductivity measurements. This model was validated to have broader applicability than the Kaganer model. Dan, Zhang, and Tao [116] developed a model that builds upon the Kaganer framework but replaces the phenomenological β with physically explicit gas transport parameters and corrects the mean free path to account for molecule–solid surface collisions along with the molecule–molecule collisions. A compact closed-form expression in terms of pressure, temperature, porosity, and specific surface area was provided by a silica aerogel composite model [115,117,118]. Its validation was carried out experimentally using the hot disk transient plane source method [119]. The Bi-Tang-Tao model [120] and the Li-Zhu-Zhao model [121] both address a fundamental limitation of the single-pore-size models above by incorporating pore size distributions. The Bi-Tang-Tao model discretizes the pore size distribution as a log-normal function and sums the Knudsen-corrected conductivity contributions from each pore size class, weighted by its fractional porosity, and was validated against six distinct aerogel systems, including silica, carbon, fumed silica, xonotlite, and RF aerogels. The Li-Zhu-Zhao model similarly decomposes the total gaseous conductivity as a porosity-weighted sum over pore classes but introduces a correction factor $C_{1}$ into the molecular mean free path term to account for molecule–solid collisions more accurately, and was validated against experimental data for silica, RF, and fumed silica aerogels.

Unlike solid-phase models, gas-phase conductivity models based on the Knudsen framework are substantially more transferable across aerogel families. The Knudsen suppression mechanism depends on the ratio of molecular mean free path to pore size, and is largely independent of solid chemistry; it applies equally to silica, carbon, RF, and biopolymer aerogels, provided pore size and porosity are known. Differences in specific surface area and pore size distribution between aerogel types do shift the characteristic pressure at which Knudsen suppression becomes significant, but the functional form of the models remains applicable. The primary limitation of transferability in this section is the accommodation coefficient, which is surface-chemistry-dependent and has been characterized primarily for silica surfaces.

Since the gaseous thermal conductivity of an aerogel cannot be measured in isolation from a single experiment, the gaseous contribution is extracted by subtracting the low-pressure baseline, representing solid and radiative conduction, from the pressure-dependent total. Table 5 summarizes the experimental techniques used to calculate the gaseous thermal conductivity in aerogels. The Transient Hot Strip (THS) method [124] embedded a thin Ni80Cr20 resistive strip within a packed powder sample inside a vacuum furnace, recording thermal conductivity from room temperature up to 900 K over a pressure range of 1 Pa to atmospheric pressure. The gaseous contribution is isolated by suppressing gas conduction below ~20 Pa and subtracting this baseline from higher-pressure values, with a reported measurement uncertainty below 3% at ambient conditions. The interlaboratory comparison study on nanoporous polyurethane aerogel [125] was unique in employing multiple laboratories and multiple techniques simultaneously, including guarded hot plate (GHP), heat flow meter (HFM) [126], transient hot wire (THW) [127,128], THS, and transient plane source (TPS) [119] methods. Zhang et al. [129] performed a TPS experiment using a custom-built apparatus capable of spanning an exceptionally wide pressure range of 0.001 Pa to 1 MPa on a nanoporous silica composite (Super-G) at 297 K, repeatedly evacuating samples to remove moisture before introducing nitrogen at controlled pressures, and they computed the gaseous conductivity directly by subtracting the vacuum thermal conductivity from the effective thermal conductivity at different pressures. Dan et al. [116] used the TPS method over a range of pressure values, and independently estimated solid and radiative contributions rather than treating their sum as the vacuum baseline. These contributions were subtracted from the effective thermal conductivity values at different pressures to estimate the gaseous thermal conductivity of two nanoporous materials. A transient hot-wire calorimetric technique [16,22,115] placed a platinum hot wire between two matched aerogel blocks in a vacuum chamber, and the temperature rise in the wire as a function of *ln* (time) yielded the thermal conductivity, with the gaseous component extracted from the difference between evacuated and pressurized measurements. Another similar transient hot-wire method [130], applied to silica aerogel plates opacified with carbon particles to suppress radiation, was used to estimate the gaseous thermal conductivity. An experiment conducted by Reichenauer et al. [17] employed three complementary techniques, hot-wire, guarded hot plate, and laser-flash [131], across a range of porous materials, including monolithic silica aerogels, granules, fumed silica powder, carbon aerogels, polyurethane foam, and packed glass spheres, fitting the measured pressure-dependent curves to a Knudsen gas conduction model to extract the gaseous contribution. Wiener et al. conducted an experiment that directly addressed high-temperature gas-phase conduction by applying the laser-flash technique to carbon aerogels [132]. The guarded hot plate approach was applied to silica aerogels and extended to a higher range of temperatures by Heinemann et al. [25], thus providing a broader range of thermal conductivity values across temperatures. Spagnol et al. [133] conducted a guarded thin-film-heater method, which used a thin electrical heater film sandwiched between two identical aerogel samples connected to cooled brass plates inside a vacuum chamber. Thermal conductivity was calculated from the measured heat flux, sample thickness, and temperature difference between the hot film and the cold plates. Swimm et al. [134] employed a transient hot-wire method that applied measurements over the wide pressure range, 10 Pa to 10 MPa, using both a vacuum chamber and a high-pressure autoclave on organic aerogel samples in argon and helium environments. The rise in measured thermal conductivity was attributed to gaseous heat conduction in the pores, and the gaseous thermal conductivity was extracted by suppressing conduction and radiation.

**Figure 5:**
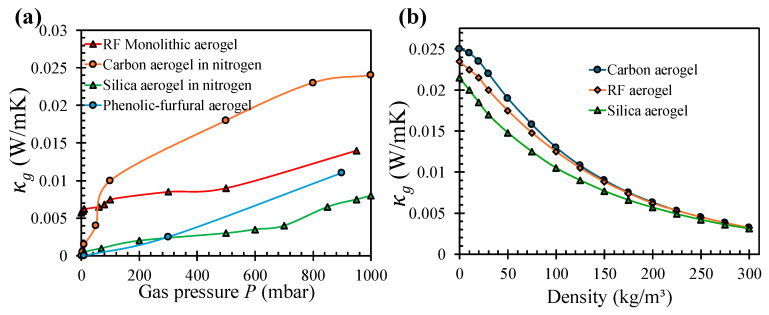
(**a**) Gaseous thermal conductivity plotted against gas pressure (Data from [22,135,136]); (**b**) gaseous thermal conductivity plotted against density for different aerogels (Data from [137]). Data compiled from multiple literature sources with differing measurement conditions; values are not strictly comparable and illustrate general trends.

Figure 5a shows the gaseous thermal conductivity $\kappa_{g}$ as a function of gas pressure for four aerogel types: RF monolithic, carbon, silica, and phenolic-furfural aerogels. All of the aforementioned aerogels exhibit the characteristic Knudsen suppression behavior, where $\kappa_{g}$ rises steeply at low pressures when molecular-regime transport transitions toward the continuum regime, and then progressively saturates as it approaches atmospheric pressure. The notably higher gaseous conductivity of carbon aerogel in nitrogen across the entire pressure range reflects its larger mean pore size relative to silica aerogel, which shifts the Knudsen transition to higher pressures and allows more gas-phase heat transfer at intermediate pressures. Figure 5b complements this by showing $\kappa_{g}$ as a function of bulk density at atmospheric pressure for carbon, RF, and silica aerogels, demonstrating a consistent and steep decline in gaseous conductivity with increasing density across all three aerogels, which is a direct consequence of the reduction in mean pore size and increase in specific surface area that accompany densification, both of which intensify Knudsen suppression. The convergence of all three curves at higher densities suggests that, beyond a critical density, the gaseous contribution becomes largely material-independent and is governed primarily by pore confinement geometry rather than chemical composition.

The theoretical models have a number of recurring limitations that constrain the predictive reliability of existing frameworks. Perhaps the most significant shortcoming is the reduction in pore geometry to a single representative diameter. Such a representation fails to capture the inherently polydisperse and hierarchical nature of aerogel pore networks. Even models that incorporate pore size distributions tend to treat contributions from individual pore classes as independent and linearly additive. As a result, the effects of features that arise due to the movement of gas molecules through differently sized pores, namely inter-pore correlations, network tortuosity, and re-entry effects, are neglected, although they could result in a change in the effective mean free path. Additionally, most models treat the gas and solid phases as thermally independent, neglecting the gas–solid coupling effect. MD studies have shown that this effect can account for a non-trivial fraction of total conductivity for moderate densities at ambient pressure. Most models were developed and typically validated against ambient temperature and a limited pressure range, with no extrapolation to elevated temperatures, cryogenic conditions, or alternative gas environments. Finally, the structural inputs, such as specific surface area, pore size distribution width, and apparent density, themselves are susceptible to marked uncertainty in their measurement and depend on the characterization technique employed. Addressing these limitations will require a concerted effort to develop frameworks that couple realistic network topology with first-principles or experimentally derived surface interaction parameters, validated across a broad and systematically varied set of aerogel compositions and environmental conditions. Experimental approaches also have several pervasive limitations that constrain the reliability and comparability of reported gaseous thermal conductivity values. The most fundamental issue is that none of the experiments measure the gaseous contribution directly, since all of them rely on an indirect subtraction strategy in which the low-pressure baseline is assumed to represent solid and radiative conduction exclusively. This assumption is only valid if the vacuum achieved is sufficient to fully suppress gas conduction. At higher pressures, residual gas conduction may be non-negligible in aerogels with larger pores, causing the baseline to be overestimated and the extracted gaseous contribution to be correspondingly underestimated. A related concern is the assumption that the solid and radiative contributions remain constant as gas pressure is varied. In practice, the presence of gas molecules can modify the effective phonon scattering environment near pore surfaces through gas–solid coupling. This means that $\kappa_{s}$ measured under vacuum may not be identical to $\kappa_{s}$ at atmospheric pressure, introducing a systematic error rarely addressed by experimental techniques. Steady-state methods such as the guarded hot plate require long equilibration times during which moisture adsorption or desorption from the aerogel surface can alter the pore gas composition and conductivity, especially at low pressures. In addition to that, the type of gas has been demonstrated to have a substantial effect on the Knudsen suppression curve, as evidenced by the comparatively few studies that employ argon or helium as the pressurizing gas, as opposed to the commonly used nitrogen. Experimental determination of thermal conductivity in aerogels is typically performed using standardized methods originally developed for bulk insulation materials, including guarded hot plate (ASTM C177 [138], ISO 8302 [139]), heat flow meter (ASTM C518 [140]), and transient techniques such as the transient plane source method (ISO 22007-2 [141]). While these standards provide a consistent framework for measurement, their underlying assumptions are not always fully satisfied in highly porous, semi-transparent, and low-density aerogel systems. Several assumptions embedded in these standards become fragile when applied to aerogels. First, radiative heat transfer, typically neglected or treated as a correction in standard methods, can contribute non-negligibly due to the partial infrared transparency of aerogels. Second, interfacial thermal contact resistance can introduce significant uncertainty because of the low stiffness and poor surface conformity of aerogel samples. Third, gas-phase conduction is strongly pressure-dependent, meaning that measurements performed under ambient conditions may not represent intrinsic material behavior. Additionally, humidity and gas composition can alter effective thermal conductivity, yet are often not controlled or reported. Finally, the extremely low thermal conductivity and small specimen thickness typical of aerogels challenge the sensitivity limits and boundary assumptions of standard measurement techniques. Finally, the vast majority of experiments are conducted at or near room temperature. This leaves the temperature dependence of the gaseous contribution, which is critical for high-temperature insulation applications, largely uncharacterized. To address these limitations, it will require standardized measurement protocols and an independent verification of the solid and radiative baseline contributions through complementary techniques. Another avenue to be addressed is the lack of systematic studies of temperature and gas-species dependence across a wider range of aerogel compositions.

### 3.3. Gas–Solid Coupling Contribution to Thermal Conductivity in Aerogels

Beyond the independently treated solid and gaseous contributions discussed before, a physically distinct and often underestimated mechanism arises from the intimate geometrical proximity of the solid nanoparticle network and the gas confined within inter-particle gaps, called the gas–solid coupled conduction, $\kappa_{c}$. At room temperature, the standard practice of breaking aerogel thermal conductivity into separately modeled solid, gas, and gas–solid coupling contributions is not entirely straightforward, since the coupling term, in many cases, is simply absorbing the shortcomings of the solid and gas models rather than representing any distinct physical mechanism. This coupling effect originates from heat transfer paths that traverse both the solid and the gas phases simultaneously. The magnitude of this contribution depends sensitively on gap geometry, pore size relative to the molecular mean free path, and the thermal accommodation coefficient at the gas–solid interface, and it cannot be captured by summing independently computed solid and gas conductivities. Table 6 gives a list of the theoretical models that have been used to calculate the gas–solid coupled contribution to thermal conductivity. The Zhao model [142] represents the aerogel as aggregates of porous secondary particles, discretizing the inter-particle gap region into cylindrical elements with gas transport described by a modified Knudsen relation, and incorporating a quasi-lattice vibration term to account for enhanced energy exchange in very narrow gaps. Solid, gas, and coupling contributions are combined in a parallel-series thermal resistance framework and validated against three independent experimental datasets [25,115,134]. The Guo-Tang model [28] decomposes coupled conduction into two channels, namely an in-plane contribution representing direct heat transfer from nanoparticles into adjacent pores, and a cross contribution representing heat bridging through chains of alternating particles and gas-filled gaps, assembled as a porosity-weighted sum and validated against resorcinol-formaldehyde, carbon, silica, and fumed silica aerogel data. The Bi-Tang model [27] takes a more compact approach, arriving at $\kappa_{c}=\frac{2}{3}\kappa_{gs}$, where $\kappa_{gs}$ is derived from a logarithmic mixing rule requiring only mean pore diameter, particle diameter, porosity, and bulk phase conductivities as inputs, validated against hot-plate measurements and the literature data for silica aerogels [143], carbon aerogels [144], and RF aerogels [145]. Lastly, the Fu D2Q9 model [146] departs from analytical frameworks entirely by reconstructing the stochastic aerogel microstructure using a random generation-growth algorithm and simulating coupled gas–solid heat transfer using the D2Q9 Lattice Boltzmann method, computing the coupled conductivity directly from the resolved heat flux field without any a priori assumptions about gap geometry and was validated against experimental data from the literature [27,123,143].

The most fundamental difficulty in quantifying the gas–solid coupling contribution is that the coupled contribution $\kappa_{c}$ is not independently measurable. It cannot be isolated experimentally in the same way that $\kappa_{s}$ and $\kappa_{g}$ can be suppressed by evacuation or opacification, because the coupling mechanism is intrinsically active whenever both phases are present simultaneously. As a result, all existing model validations rely on comparisons with total effective conductivity measurements, from which the coupled contribution is inferred only after subtracting separately modeled solid and radiative terms, and any errors in those independent estimates propagate directly into the apparent validation of the coupling model. This makes it genuinely difficult to assess whether a given coupling model is physically correct. Additionally, the analytical models in this family share a reliance on idealized geometric representations of the inter-particle gap that bear little resemblance to the disordered, polydisperse, and fractal geometry of real aerogel contact zones. The assumption of a single representative gap height or contact length is particularly problematic, given that the coupling contribution is extremely sensitive to gap geometry, which could translate to large changes in the local Knudsen number and, therefore, in the local gas conductivity within the gap. More broadly, none of the existing models account for the temperature dependence of the coupling contribution, the role of surface chemistry in modifying the accommodation coefficient at the gas–solid interface, which is likely to be significant in real-world operating conditions. The reliable quantification of the coupled conduction mechanism will ultimately require a combination of nanoscale experimental probes and atomistic or mesoscale simulations that can resolve gas–solid coupling at the length scales where it physically occurs.

The Bi-Tang model [27], the most experimentally validated coupling model reviewed here, was explicitly tested against silica, carbon, and RF aerogel datasets, providing a degree of cross-material validation not present in the solid or gas models individually. However, the geometric assumptions embedded in the coupling models, specifically the idealized particle-pore geometry, remain anchored in particle-aggregated morphologies. For fibrillar aerogels, where the solid–gas interface geometry is fundamentally different, direct application of these models is not appropriate without structural re-parameterization. The coupling contribution is likely less significant in high-conductivity carbon aerogels where solid conduction dominates, but remains an important term in low-density biopolymer aerogels where solid and gas contributions are more comparable in magnitude.

### 3.4. Total Thermal Conductivity in Aerogels

**Figure 6:**
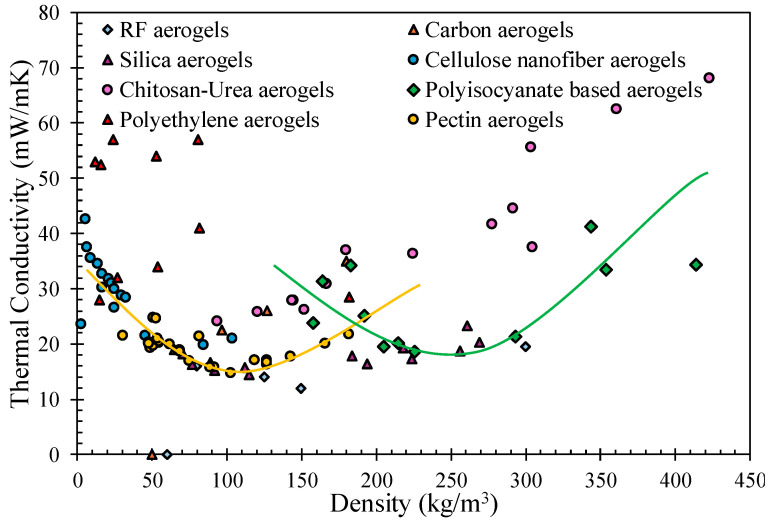
Plot of total thermal conductivity against density for different aerogels. Data from [137,147,148,149,150,151,152,153]. The two lines in the figure highlight the non-monotonic trends in the presented datasets.

The complexity of the total thermal conductivity as a function of density across different types of aerogels is illustrated in Figure 6. As captured by the two trend lines (for the pectin and the polyisocyanate-based aerogels), total conductivity initially decreases with increasing density before reaching a minimum and rising again. This is a direct consequence of competing density dependences, i.e., at low densities, the gaseous and radiative contributions dominate and decrease as pore sizes shrink and Knudsen suppression intensifies, while at higher densities, the solid conduction contribution scales steeply with solid volume fraction and drives the total back upward. The two trend lines reflect the fact that the density at which this minimum occurs is material-specific, shifting with composition, pore size distribution, and backbone chemistry. The substantial scatter in reported values at any given density further confirms that density alone is an insufficient predictor of thermal performance, and that the anomalously high values seen for chitosan-urea and polyethylene aerogels underscore the role of material chemistry as a variable that shifts the entire conductivity–density relationship. This has already been demonstrated by Malfait et al. [49], while making a call to action about comprehensively understanding and validating the methods, as well as being aware of the limitations of their application to specific materials and sample sizes.

While the additive decomposition in Equation (1) is widely adopted for its analytical convenience, the critical discussions in the preceding sections collectively reveal that it rests on an assumption of independence between contributions that is not strictly satisfied in real aerogel systems. The solid conductivity is suppressed relative to its bulk value by phonon constriction at inter-particle necks and by nanoscale size effects, but its magnitude depends on the same pore geometry and particle contact arrangement that govern the coupling contribution. The two cannot be independently tuned without simultaneously modifying the other. The gaseous contribution is Knudsen-suppressed by pore confinement, but its effective value at ambient pressure is augmented by the gas–solid coupling mechanism. So, $\kappa_{g}$ and $\kappa_{c}$ cannot be treated as separate additive terms. Experimental determination of the total conductivity is comparatively straightforward using methods like guarded hot plate, transient hot wire, and TPS, all of which yield reliable bulk values. Yet, the decomposition of that measured total into its constituent contributions remains ambiguous, as no single experiment can independently resolve all three terms simultaneously. The distribution of thermal conductivity values at similar densities is not just a materials problem. It is a result of structural variability, but it also comes down to the inconsistencies in experimental measurements and the different reporting practices, making comparisons harder than they should be. The consequence is that validated total conductivity models are often the product of compensating errors between individual component models rather than independently verified physical descriptions, and reported agreements between model and experiment should therefore be interpreted with suitable caution. A robust predictive framework for total aerogel thermal conductivity must therefore move beyond the strict additive assumption and toward coupled, multi-scale treatments that resolve the interactions between solid phonon transport, gas molecular dynamics, and inter-phase energy exchange within a unified structural description of the aerogel network.

While numerous models have been developed to describe thermal transport in aerogels, their applicability depends strongly on the level of structural detail required and the intended use of the model. Effective medium approaches offer simplicity and require minimal input parameters, making them suitable for rapid estimation and engineering-scale calculations, but they lack the ability to capture nanoscale structural effects and often rely on empirical fitting. Percolation and fractal models provide improved representation of connectivity and structural scaling, particularly at high porosity, but remain limited by their reliance on idealized geometries. Phonon-based and size-effect models are more physically grounded at the nanoscale and can capture boundary scattering effects, yet require detailed knowledge of structural length scales that are not always experimentally accessible. More advanced approaches, such as pore-network or multi-scale simulations, offer higher fidelity by incorporating realistic geometries, but at the cost of increased computational complexity and input requirements. As a result, model selection should be guided by the trade-off between simplicity and physical fidelity, with simpler models appropriate for trend analysis and design screening, and more detailed models required for predictive accuracy and structure-specific investigations.

In addition to pressure and temperature, environmental humidity plays a critical role in aerogel thermal transport [154,155]. The presence of water vapor within nanopores modifies gas-phase conduction due to differences in thermophysical properties between humid air and dry air. Furthermore, adsorption of moisture on pore surfaces or capillary condensation within nanoscale pores can partially suppress the Knudsen effect by increasing effective collision frequency and reducing gas–wall scattering dominance. This leads to an increase in gaseous thermal conductivity under humid conditions. Moisture adsorption may also influence interfacial thermal resistance and gas–solid coupling mechanisms, particularly in hydrophilic aerogels such as silica and cellulose-based systems. As a result, variations in ambient humidity can contribute to discrepancies across experimental datasets and should be carefully controlled or reported in both measurements and modeling frameworks. But humidity is often not consistently controlled or reported in experimental studies, contributing to the lack of comparability across datasets.

The aggregation of thermal conductivity data across the aerogel literature is complicated by significant inconsistencies in measurement methodology and reporting practices. Malfait et al. [49] compiled over 500 data points from 87 studies and demonstrated that a substantial fraction of reported values are either physically implausible or insufficiently documented, highlighting a systemic reliability issue in the field. In the present review, the datasets presented in Figure 4, Figure 5 and Figure 6 and Table 2, Table 4 and Table 6 are not normalized to a common set of experimental conditions (temperature, pressure, gas composition, humidity, or sample geometry), primarily because such information is not consistently reported in the source literature. As a result, these datasets should be interpreted as indicative of general trends rather than directly comparable quantitative benchmarks. The observed scatter in reported thermal conductivity values, therefore, reflects not only genuine structural variability between aerogel systems but also interlaboratory differences in measurement technique, sample preparation, and environmental control. This lack of comparability fundamentally limits the validation of theoretical models and complicates the extraction of reliable structure–property relationships. Addressing this issue will require the adoption of standardized experimental protocols and comprehensive reporting practices, including full disclosure of measurement conditions and structural characterization parameters.

## 4. Conclusions

This review examines the structural features, heat transfer mechanisms, and modeling approaches that enable ultra-low thermal conductivity in nanoporous aerogels. Their superior insulation arises from the coupled action of the interacting processes of solid-phase phonon conduction, Knudsen-suppressed gas conduction, and gas–solid coupled conduction. These mechanisms cannot be fully isolated either experimentally or theoretically. Solid conduction is strongly reduced below bulk values by phonon constriction at inter-particle necks and nanoscale effects. However, analytical models typically account for fractal geometries and single representative pore sizes while ignoring the effect of polydispersity. Gaseous conduction follows the Knudsen regime. Even though modeling has improved from single-pore approximations to pore-size-distribution-aware frameworks, there has been no resolution for the incorporation of accommodation coefficients, gas-specific effects, and gas–solid coupling. Experimentally, reliance on indirect pressure-dependent subtraction methods introduces systematic errors, and interlaboratory variability largely reflects methodological differences rather than material differences. Gas–solid coupling remains the least characterized term, since it is not directly measured or validated, and is geometrically oversimplified in models. Therefore, it requires particular scrutiny. Rather than representing a distinct heat transfer channel, it frequently compensates for deficiencies in the solid and gas models. This occurs especially where confinement, interfacial scattering, and gas–surface interactions act simultaneously and cannot be correctly partitioned. These modeling limitations are compounded by poor experimental standardization, and discrepancies between models and experiments cannot be attributed to theory alone [49]. Total effective conductivity shows a characteristic non-monotonic dependence on density due to competing trends among the individual mechanisms. Density alone does not capture pore connectivity, neck geometry, tortuosity, or pore-size distribution, which are all strongly involved in heat conduction through aerogels. In future studies, multi-scale models linking atomistic phonon transport to realistic mesoscale networks would be required in order to determine the gas–solid coupling contribution to the total effective thermal conductivity. Along with this, standardized experimental protocols with comprehensive structural characterization are necessary to make reliable comparisons.

- The widespread use of an additive thermal conductivity decomposition in aerogels has outpaced the physical justification for treating its individual terms as independent and separately measurable.
- The persistent introduction of a third “coupling” contribution is a symptom that current solid and gas models are incomplete, not proof that the conductivity can always be partitioned into three clean channels.
- Density and porosity are useful screening parameters, but they fail as general predictive variables because they do not encode pore connectivity, neck geometry, or pore-size polydispersity.
- The observed V-shaped conductivity–density behavior across aerogel families is direct evidence that one-parameter descriptions are inadequate.
- A physically meaningful design framework must treat aerogel conductivity as a structure-sensitive, multi-scale transport problem rather than a fitted sum of nominal phase contributions.

The mechanisms and models reviewed here were developed predominantly within the silica aerogel literature, which provides the most mature experimental and modeling foundation. However, the underlying physics, Knudsen-suppressed gas conduction, phonon constriction at inter-particle contacts, and gas–solid interfacial energy exchange are not silica-specific. Gas-phase models transfer most directly across aerogel families, as they depend on pore geometry rather than solid chemistry. Solid-phase models transfer qualitatively to other particle-aggregated systems such as RF and carbon aerogels but require re-parameterization for fibrillar systems. Coupling models occupy an intermediate position: the Bi-Tang framework has been validated across silica, carbon, and RF aerogels, but its geometric assumptions limit direct application to fibrillar morphologies. Where conclusions in this review are drawn from silica-dominated datasets, they should be interpreted as establishing the framework rather than universal quantitative predictions. As highlighted by Malfait et al. [49], the lack of standardized measurement and reporting protocols introduces systematic uncertainty into the literature, meaning that discrepancies between models and experiments cannot be attributed to theory alone.

Emerging aerogel architecture based on 1D and 2D building units further reinforces the need for structure-aware models, as they introduce fundamentally different transport pathways compared to classical nanoparticle networks. The emergence of anisotropic aerogels further underscores that thermal transport must be treated as a direction-dependent, structure-sensitive phenomenon rather than a scalar material property. Unreported environmental factors such as humidity further contribute to the variability and limited comparability of thermal conductivity data across the literature. Ultimately, next-generation aerogel insulators will require moving beyond density optimization to simultaneous targeted engineering of pore-size distribution, backbone connectivity, and surface chemistry, guided by the structure–property relationships outlined here, to minimize all heat transfer pathways in concert. Radiative heat transfer, while important under high-temperature conditions, is not treated in detail here, as the present review focuses on conduction-dominated transport mechanisms.

## Figures and Tables

**Table 3:** Model and Experimental Method comparison.

**Analytical Model**	**Model Type**	**Advantage**	**Limitation**	**Application**
	Effective medium	Simple, fast	Oversimplified	Screening
	Percolation/Fractal	Captures connectivity	Idealized	High porosity systems
	Phonon-based	Physically grounded	Needs structural data	Nanoscale analysis
	Pore-network	High accuracy	Complex	Detailed prediction
**Experiment**	**Method**	**Advantage**	**Limitation**	**Application**
	Guarded hot plate	High accuracy	Large samples	Bulk validation
	Heat flow meter	Simple	Calibration limits	Moderate κ materials
	TPS (Hot Disk)	Small samples, fast	Contact sensitivity	Nanostructured aerogels

**Table 4:** Theoretical models for the calculation of gaseous thermal conductivity of aerogels.

Model	How It Was Developed	Physical Parameters	Validation
**Kaganer model** [30] $\kappa_{g}=\frac{\kappa_{g}^{0}}{1+2\beta Kn}$	Closed-form Knudsen correction	$\kappa_{g}^{0}$ (free-gas thermal conductivity), β (accommodation + adiabatic-coefficient-dependent constant), Kn (mean free path/pore diameter; mean free path depends on molecule diameter, T, p)	Baseline model
**Zeng gas-kinetics model** [16,115] $\kappa_{g}=\frac{\left(2.25\gamma -1.25\right)461\left(\frac{p_{g}}{k_{B}T}\right){\left(\frac{8k_{B}T}{m_{g}\pi }\right)}^{0.5}C_{V}}{\left(\frac{0.25S\rho_{a}}{\phi }\right)+\sqrt{2}\left(\frac{p_{g}}{k_{B}T}\right)\pi d_{g}^{2}N_{A}}$	Analytical solution based on gas kinetics tailored to porous media, aiming to correct mean-free-path treatment in pores	γ (adiabatic coefficient of the gas), $m_{g}$ (molecular mass of the gas), $N_{A}$ (Avogadro’s constant), $C_{V}$ (constant volume heat capacity), $\rho_{a}$ (density of the aerogel), ϕ (porosity), S (specific surface area). Both ϕ and S can be calculated from the pore size and the solid particle diameter of the aerogel [122]	Validated against the Kaganer baseline model
**Reichenauer model** [17] $\kappa_{g}=\frac{\kappa_{g}^{0}\phi }{1+2\beta Kn}$ → $\kappa_{g}=\frac{\kappa_{g}^{0}\phi }{1+2\beta \left(\frac{\kappa_{g}^{0}}{D}\right)\left(\frac{p_{0}}{p}\right)}$	Classical kinetic gas theory acknowledging that gas thermal conductivity inside pores depends on how often molecules collide with each other vs. the pore walls.	$\kappa_{g}^{0}$ (free-gas thermal conductivity at reference pressure), ϕ (overall porosity), β (pore wall accommodation + adiabatic-coefficient-dependent constant), Kn (Knudsen number = mean free path/effective pore diameter)	Validated the model by comparing it to measured thermal conductivity vs. gas pressure curves for a wide range of porous materials (e.g., fumed silica, aerogels, foams) with pore sizes from tens of nanometers up to micrometers [123].
**Bi-Tang-Tao model** [120] $\kappa_{g}=\sum_{i=1}^{n}\phi_{i}K\left(D_{i}\right)$ where $\phi_{i}\approx \frac{\Delta D}{\sqrt{2\pi }\sigma }exp\left(-\frac{{\left(D_{i}-D\right)}^{2}}{2\sigma^{2}}\right)$	Pore size distribution introduced in the Knudsen gas conduction method.	Gas properties: $\kappa_{g}^{0}$ (free gas thermal conductivity), $d_{g}\;$(molecular diameter), $m_{g}\;$(molecular mass), $C_{v}$ (heat capacity), γ (adiabatic coefficient), $p_{g}$ (gas pressure), T (temperature). Structural properties: D (mean pore diameter), σ (pore size distribution width, ϕ (porosity), S (specific surface area), $\rho_{a}$ (aerogel density), $\rho_{p}$ (skeletal density)	Compared predictions against experimental data for six aerogel systems: Silica aerogel (air), carbon aerogel (Ar), fumed silica aerogel, Xonotlite aerogel, and organic RF aerogel.
**Dan-Zhang-Tao model** [116] $\kappa_{g}=\frac{\kappa_{g}^{free}}{1+\frac{2\gamma }{\mathrm{Pr}\left(\gamma +1\right)}\frac{2\alpha }{\alpha +1}Kn\;\;}$ where: $\kappa_{g}^{free}=\kappa_{g}^{0}=\left(2.25\gamma -1.25\right)\eta C_{v}$ Dynamic viscosity: $\eta =0.461n_{g}m_{g}{\left(\frac{8k_{B}T}{\pi m_{g}}\right)}^{\frac{1}{2}}l_{m}$ Mean free path: $l_{m}=\frac{1}{\sqrt{2}n_{g}\pi d_{g}^{2}+0.25S_{s}\rho_{por}/\phi }\;$ Number density: $n_{g}=\frac{p}{k_{B}T}$	Kaganer’s Knudsen model corrected via kinetic theory substitution for mean free path and gas conductivity, yielding explicit pore-size-dependent gaseous thermal conductivity expression.	Gas parameters: $\kappa_{g}^{0}$ (free gas thermal conductivity), $p_{g}$(gas pressure), T (temperature), $m_{g}\;$(molecular mass), $d_{g}\;$(molecular diameter), $C_{v}$ (heat capacity), γ (adiabatic coefficient), Pr (Prandtl number), α (accommodation coefficient). Structural parameters: $S_{s}$ (specific surface area), ϕ (porosity), $\rho_{por}$ (apparent density of aerogel)	Compared with decomposed experimental data and measured effective thermal conductivity from 0.01 Pa to 1 MPa.
**Silica Aerogel composites model** [115,117,118] $\kappa_{g}=\frac{60.22\times pT^{-0.5}}{0.25S_{a}\phi_{a}+4.01\times 10^{-4}pT^{-1}\;\;}$	Kinetic theory foundation with porosity-modified phonon mean free path combined into a closed-form gaseous thermal conductivity expression.	Gas properties: p (pressure), T (temperature). Structural parameters: $\phi_{a}$ (porosity), $S_{a}$ (specific surface area)	Validated experimentally via hot disk transient plane source method [119], with effective thermal conductivity decomposed into coupled solid–gas and radiative contributions.
**Li-Zhu-Zhao model** [121] $\kappa_{g}\left(D\right)=\sum_{i=1}^{n}\phi_{i}\kappa_{g}\left(D_{i}\right)$ where: $\kappa_{g}\left(D_{i}\right)\approx \frac{\kappa_{g0}}{1+\frac{2C_{1}l}{D_{i}}}$	Kinetic theory foundation with Zeng model [115] mean free path modified by a correction factor for molecule–solid collisions, reformulated into closed-form Knudsen-like expression.	$\kappa_{g0}$ (free gas conductivity), $C_{1}$ (Correction coefficient which modifies the mean free path to include the molecule–solid collision term), i (pore index), n (number of aerogel pores), $D_{i}$ (mean pore diameter of pore with index i), $\phi_{i}$ (porosity contribution to total porosity of pore with index i)	DLCA-reconstructed 3D aerogel subjected to DSMC simulations as benchmark; correction factor fitted by matching model to DSMC, and then validated against experimental gaseous thermal conductivity data from silica aerogels [27], RF aerogels, and fumed silica aerogels [17].

**Table 5:** Experimental techniques to capture the gaseous thermal conductivity of aerogels.

Experiment	Gas Contribution	Bulk Thermal Conductivity	Reported Range
**Transient Hot Strip (THS) method** [124] Effective thermal conductivity measured via Transient Hot Strip (THS) method on packed powder in vacuum furnace, covering 1 Pa to atmospheric pressure and room temperature to 900 K, with uncertainty less than 3% at ambient conditions.	THS method used over 1 Pa to atmospheric pressure; gaseous contribution isolated by subtracting low-pressure baseline (solid + radiative only, below ~20 Pa) from total conductivity at higher pressures.	0.46 W/mK	~0 to ~0.02 W/mK
**Nanoporous polyurethane (PU) aerogel experiment** [125] Interlaboratory comparison measuring total effective thermal conductivity of nanoporous polyurethane aerogel panels using stationary (GHP, HFM) and transient (THW, THS, TPS) methods at 20–60 °C, corrected to reference atmospheric pressure of 1013 hPa.	Gaseous contribution isolated via guarded-hot-plate measurements as a function of nitrogen pressure; vacuum baseline (solid + radiative only) subtracted from higher-pressure values, with corrections for atmospheric pressure variations.	0.2 W/mK	~0 to ~0.011 W/mK
**Zhang’s Transient Plane Source (TPS) method** [129] TPS method measuring effective thermal conductivity of nanoporous silica composite (Super-G) over 0.001 Pa to 1 MPa at 297 K, with nitrogen pressure gradually increased after repeated vacuumization to remove moisture and residual gases.	Gaseous contribution derived from TPS pressure-dependent measurements by subtracting ultimate vacuum conductivity (solid + radiation only, ~0.001 Pa) from total conductivity at each nitrogen pressure up to 1 MPa.	1.34 W/mK	~0.03 to ~0.035 W/mK
**Dan’s Transient Plane Source (TPS) method** [116] TPS method (Hot Disk TPS2500S) measuring effective thermal conductivity of two nanoporous materials over 0.01 Pa to 1 MPa nitrogen pressure at 297 K, with repeated evacuation below 0.01 Pa to remove adsorbed gases before each measurement.	Gaseous contribution isolated by subtracting high-vacuum baseline (solid + radiative only) from total conductivity at each nitrogen pressure level across the full pressure range.	1.34 W/mK	~0 to ~0.034 W/mK
**Transient hot-wire calorimetric technique** [16,22,115] Transient hot-wire method measuring thermal conductivity of monolithic aerogel blocks over ~10^−4^ mbar to 1 bar gas pressure at controlled temperatures, with platinum wire embedded between two identical aerogel samples.	Gaseous contribution isolated by subtracting near-vacuum baseline (solid + radiation only) from total conductivity at increasing pressures up to atmospheric, with the pressure-dependent rise attributed entirely to gas conduction.	~1.3–1.4 W/mK	~0.005 to ~0.008 W/mK
**Transient hot-wire thermal conductivity method** [130] Transient hot-wire method measuring thermal conductivity of carbon-opacified silica aerogel plates over ~40 mTorr to 760 Torr gas pressure, with platinum wire embedded in grooves between sample plates and conductivity derived from slope of temperature rise vs. ln(time).	Gaseous contribution isolated by subtracting low-pressure baseline (solid + radiative only, ~10^−3^ atm) from total conductivity at each higher pressure, with the pressure-dependent conductivity rise attributed entirely to gas-phase heat transport.	1.3 W/mK	~0.01 to ~0.013 W/mK
**Reichenauer experiment** [17] Hot-wire, guarded hot-plate, and laser-flash methods measuring thermal conductivity of silica aerogel, aerogel granules, fumed silica, carbon aerogels, porous PU foam, and glass spheres as a function of nitrogen pressure (vacuum to ~1 bar).	Gas contribution to the thermal conductivity is obtained from pressure-dependent conductivity curves fitted with Knudsen gas conduction model.	Silica aerogel (monolithic): ≈0.012–0.015 W/mK. Silica aerogel granules: ≈ 0.010–0.013 W/mK. Fumed silica powder: ≈0.020–0.025 W/mK. Carbon aerogel: ≈0.02–0.05 W/mK. Polyurethane (PU) foam: ≈0.03–0.04 W/mK. Packed glass spheres: ≈1.0–1.4 W/mK.	Silica aerogel (monolithic): =0 to ~0.01 W/mK. Silica aerogel granules: =~0.007 to ~0.01 W/mK. Fumed silica powder: =0 to 0.015 W/mK. Carbon aerogel: =~0.01 to ~0.012 W/mK. Polyurethane (PU) foam: =~0.015 to ~0.026 W/mK. Packed glass spheres: =~0.02 to ~0.026 W/mK.
**Laser-flash technique for Carbon aerogels** [132] Laser-flash technique measuring thermal conductivity of carbon aerogel disks at high temperatures, with front-surface laser pulse heating and back-surface infrared temperature monitoring.	Effective thermal conductivity derived from laser-flash diffusivity and DSC-measured specific heat, with measurements up to 1773 K under vacuum and 0.1 MPa argon; gaseous contribution isolated by subtracting vacuum conductivity from argon-atmosphere values.	In vacuum: ≈0.09 W/mK at 1773 K. In argon atmosphere: ≈0.12 W/mK.	≈0 to 0.02 W/mK
**Heinemann’s guarded hot plate experiment** [25] Guarded hot plate apparatus (LOLA III) measuring apparent thermal conductivity of silica aerogel over ~10^−4^ to 10^3^ hPa gas pressure and 100–650 K, with sample placed between heated and cold plates inside a vacuum chamber	Gaseous contribution isolated by subtracting low-pressure baseline (solid + radiation only, Knudsen regime) from total conductivity at each higher pressure, with the pressure-dependent rise attributed to increasing gas molecular collisions.	≈1.3–1.4 W/mK	≈0.01 to 0.02 W/mK
**Spagnol’s steady-state guarded thin-film-heater method** [133] Steady-state guarded thin-film-heater method measuring thermal conductivity of silica aerogel over ~10^−5^ mbar to atmospheric pressure, with symmetric heat flow through two identical samples and conductivity derived from heat flux, sample thickness, and temperature difference.	Gaseous contribution isolated by subtracting vacuum baseline (solid + radiative only, below ~10^−2^ mbar Knudsen regime) from total conductivity at each air pressure up to atmospheric, with the pressure-dependent rise quantifying gas-phase heat transport.	~0.008–0.021 W/mK	≈0 to 0.013 W/mK
**Swimm’s transient hot-wire thermal conductivity method** [134] Transient hot-wire method measuring effective thermal conductivity of organic aerogel over 10 Pa to 10 MPa at ~21 °C, using vacuum chamber for low pressures and high-pressure autoclave for elevated pressures, with conductivity derived from analytical hot-wire heat-transfer solution.	Gaseous contribution extracted indirectly from pressure-dependent total conductivity measurements, with near-vacuum baseline (solid + radiation only) subtracted and pressure-dependent rise attributed to pore gas conduction via theoretical model analysis.	≈0.23 W/mK	Argon free-gas conductivity: ~0.017 W/mK Helium free-gas conductivity: ~0.154 W/mK

**Table 6:** Theoretical models for the calculation of the gas–solid coupling contribution to thermal conductivity.

Model	How It Was Developed	Physical Parameters	Validation
**Zhao model** [142] $\kappa_{ce}=\frac{a}{\pi h_{gap}^{2}}\sum_{i=1}^{n}{\left(\frac{D_{i}}{\kappa_{g,i}A_{i}}+\frac{y_{i}}{\kappa_{part}A_{i}}\right)}^{-1}$ where: $y_{i}=l_{contact}+2\sqrt{h_{gap}^{2}-{\left(\frac{ih_{gap}}{N}\right)}^{2}}$	Analytical continuum model representing aerogel as porous secondary particle aggregates, combining solid, gas, and coupling conduction via parallel-series approach, with gas transport described by a modified Knudsen relation accounting for quasi-lattice molecular vibrations in narrow inter-particle gaps.	P (Aerogel porosity), D (Pore diameter), $d_{part}$ (Secondary particle diameter), $\phi_{part}$ (Particle porosity), $l_{contact}$ (Interparticle contact length), $h_{gap}$ (Particle gap height), p, T (Gas pressure, temperature), $l_{g}$ (Gas mean free path), $\kappa_{g0}$ (Free gas thermal conductivity), $d_{g},\;M$ (Gas molecular diameter, weight), $C_{v}$ (Gas specific heat), α (Energy accommodation coefficient), $\kappa_{part}\;$ (Bulk thermal conductivity), $l_{part}$ (Phonon mean free path), B (Quasi-lattice vibration parameter), $S_{a}$ (Aerogel specific surface area)	The predicted effective gaseous thermal conductivity showed good agreement with experimental data from Zeng et al. [115], Heinemann et al. [25], Swimm et al. [134].
**Guo-Tang model** [28] $\kappa_{c}=\phi_{c,in}\kappa_{c,in}+\phi_{c,cross}\kappa_{c,cross}$ where: $\kappa_{c,in}=\frac{h_{total}}{\pi \left(R^{2}-\frac{a^{2}}{4}\right)}\sum_{i=1}^{1000}{\left({\frac{\delta_{i}}{\kappa_{g,i}A}}_{i}+\frac{y_{i}}{\kappa_{p}A_{i}}\right)}^{-1}$; $h_{total}=\sqrt{4R^{2}-a^{2}}$; $y_{i}=2\sqrt{R^{2}-{\left(\frac{a}{2}+i\left(\frac{R-\frac{a}{2}}{1000}\right)\right)}^{2}}$; $\delta_{i}=h_{total}-y_{i}$; $A_{i}=\pi \left[\left(2i+1\right){\left(\frac{R-\frac{a}{2}}{1000}\right)}^{2}+a\left(\frac{R-\frac{a}{2}}{1000}\right)\right]$ $\kappa_{c,cross}=\frac{D+2R}{R}\times \frac{2\kappa_{p}\kappa_{g}}{\kappa_{g}-\kappa_{p}}\left[1-\frac{\kappa_{p}\left(D+2R\right)}{2R\left(\kappa_{g}-\kappa_{p}\right)}\ln\left(1+\frac{2R\left(\kappa_{g}-\kappa_{p}\right)}{\kappa_{p}\left(D+2R\right)}\right)\right]$	Two coupled heat transfer mechanisms: Local solid–gas interaction ($\kappa_{c,in}$)—Heat transfers happen from solid nanoparticles into adjacent gas-filled pores. Thermal-bridge interaction ($\kappa_{c,cross}$)—Heat transfers happen through chains of adjacent particles and gas confined in the narrow gaps between particles.	ϕ (Porosity), R (Particle radius), d (Particle diameter), D (Pore diameter), a (Particle contact length), $\kappa_{g}$ (Gas thermal conductivity), $d_{g}$ (Gas molecular diameter), l (Gas mean free path), $\kappa_{p}$ (Particle thermal conductivity)	Validated by comparing predictions with experimental measurements for several aerogel systems, including resorcinol-formaldehyde aerogels, carbon aerogels, silica aerogels, fumed silica aerogels.
**Bi-Tang model** [27] $\kappa_{c}=\frac{2}{3}\kappa_{gs}$ where: $\kappa_{gs}=\frac{\frac{D+d_{p}}{d_{p}}2\kappa_{p}\kappa_{g}}{\kappa_{g}-\kappa_{p}}\left[1-\frac{\frac{D+d_{p}}{d_{p}}\kappa_{p}}{\kappa_{g}-\kappa_{p}}\ln\left(1+\frac{\frac{\kappa_{g}-\kappa_{p}}{\kappa_{p}}d_{p}}{D+d_{p}}\right)\right]\;\;$	The total heat flux is decomposed into heat along the solid backbone and heat crossing gas–solid–gas regions.	D (Mean pore size), $d_{p}$ (Particle diameter), $\kappa_{g}$ (Gas thermal conductivity), ϕ (Porosity), $\kappa_{p}$ (Particle thermal conductivity)	Validated with the hot-plate method experiment, and also with the literature data on silica aerogels [143], carbon aerogels [144], and RF aerogels [145].
**Fu’s D2Q9 model** [146] $\kappa_{c}=N_{x}\int q_{c}dy\frac{1\;}{\Delta T\int dy}$	Stochastic open-cell aerogel microstructure reconstructed via random generation-growth method, with D2Q9 Lattice Boltzmann Method simulating simultaneous solid and gas phase heat transfer to compute effective thermal conductivity from integrated heat flux across the simulation domain.	N (Number of target nodes), $q_{c}$ (Heat flux across the domain), T (Temperature)	Model validated by comparison with experimental measurements reported in the literature [27,123,143].

## Data Availability

The data that support the findings of this study are available from the corresponding author upon reasonable request.
